# Supplementary material for: The Systems Biology Research Tool: evolvable open-source software
Source: BMC Syst Biol. 2008 Jun 29;2:55. doi: 10.1186/1752-0509-2-55 (PMC2446383; doi:10.1186/1752-0509-2-55)
Supplement: Additional file 1 — SBRT Archive. An archive of the current version of the Systems Biology Research Tool. [file 1752-0509-2-55-S1.zip › sbrt-1.4.0/doc/developers_guide/api/sbrt/shell/text/math/package-tree.html]

sbrt.shell.text.math Class Hierarchy


|  |  |  |
| --- | --- | --- |
| **Overview** \| **Package** \| Class \| Use \| **Tree** \| **Deprecated** \| **Index** \| **Help** | |  |
| **PREV**   NEXT | **FRAMES**    **NO FRAMES**     **All Classes** |  |


---


## Hierarchy For Package sbrt.shell.text.math

**Package Hierarchies:**: All Packages

---

## Class Hierarchy

- java.lang.Object
  - sbrt.shell.text.math.**BoundedIntervalFormatV1** (implements sbrt.shell.text.math.IntervalFormat)- sbrt.shell.text.math.**CoeffFormatV1** (implements sbrt.shell.text.math.CoeffFormat)- sbrt.shell.text.math.**CoeffFormatV2** (implements sbrt.shell.text.math.CoeffFormat)- sbrt.shell.text.math.**CorrResFormatterV1** (implements sbrt.shell.text.math.CorrResultsFormatter<T>)- sbrt.shell.text.math.**DirEdgeFormatV1**<N> (implements sbrt.shell.text.math.DirEdgeFormat<N>)- sbrt.shell.text.math.**IndexPairFormatV1**<R,C> (implements sbrt.shell.text.math.IndexPairFormat<R,C>)- sbrt.shell.text.math.**IntervalFormatV1** (implements sbrt.shell.text.math.IntervalFormat)- sbrt.shell.text.math.**LinCombTermFormatV1**<V> (implements sbrt.shell.text.math.LinearCombTermFormat<V>)- sbrt.shell.text.math.**LinCombTermFormatV2**<V> (implements sbrt.shell.text.math.LinearCombTermFormat<V>)- sbrt.shell.text.math.**LinearCombFormatV1**<V> (implements sbrt.shell.text.math.LinearCombFormat<V>)- sbrt.shell.text.math.**LinearCombFormatV2**<V> (implements sbrt.shell.text.math.LinearCombFormat<V>)- sbrt.shell.text.math.**MatrixElementFormatV1**<R,C> (implements sbrt.shell.text.math.MatrixElementFormat<R,C,E>)- sbrt.shell.text.math.**SenseFormat** (implements sbrt.shell.text.SimpleFormat<T>)

## Interface Hierarchy

- sbrt.shell.text.**Format**
  - sbrt.shell.text.**SimpleFormat**<T> (also extends sbrt.shell.text.Formatter<T>, sbrt.shell.text.Parser<T>)
    - sbrt.shell.text.math.**CoeffFormat**- sbrt.shell.text.math.**DirEdgeFormat**<N>- sbrt.shell.text.math.**IndexPairFormat**<R,C>- sbrt.shell.text.math.**IntervalFormat**- sbrt.shell.text.math.**LinearCombTermFormat**<V>- sbrt.shell.text.math.**MathExprFormat**<E,V>
                - sbrt.shell.text.math.**LinearCombFormat**<V>
                - sbrt.shell.text.math.**MatrixElementFormat**<R,C,E>
  - sbrt.shell.text.**Formatter**<T>
    - sbrt.shell.text.math.**CorrResultsFormatter**<T>- sbrt.shell.text.**SimpleFormat**<T> (also extends sbrt.shell.text.Format, sbrt.shell.text.Parser<T>)
        - sbrt.shell.text.math.**CoeffFormat**- sbrt.shell.text.math.**DirEdgeFormat**<N>- sbrt.shell.text.math.**IndexPairFormat**<R,C>- sbrt.shell.text.math.**IntervalFormat**- sbrt.shell.text.math.**LinearCombTermFormat**<V>- sbrt.shell.text.math.**MathExprFormat**<E,V>
                    - sbrt.shell.text.math.**LinearCombFormat**<V>
                    - sbrt.shell.text.math.**MatrixElementFormat**<R,C,E>
    - sbrt.shell.text.**Parser**<T>
      - sbrt.shell.text.**SimpleFormat**<T> (also extends sbrt.shell.text.Format, sbrt.shell.text.Formatter<T>)
        - sbrt.shell.text.math.**CoeffFormat**- sbrt.shell.text.math.**DirEdgeFormat**<N>- sbrt.shell.text.math.**IndexPairFormat**<R,C>- sbrt.shell.text.math.**IntervalFormat**- sbrt.shell.text.math.**LinearCombTermFormat**<V>- sbrt.shell.text.math.**MathExprFormat**<E,V>
                    - sbrt.shell.text.math.**LinearCombFormat**<V>
                    - sbrt.shell.text.math.**MatrixElementFormat**<R,C,E>

---


|  |  |  |
| --- | --- | --- |
| **Overview** \| **Package** \| Class \| Use \| **Tree** \| **Deprecated** \| **Index** \| **Help** | |  |
| **PREV**   NEXT | **FRAMES**    **NO FRAMES**     **All Classes** |  |


---
